# Surgical management and outcomes of renal tumors with inferior vena cava extension among children: a single center retrospective study from Pakistan

**DOI:** 10.1186/s12887-024-05122-1

**Published:** 2024-10-10

**Authors:** Huma Faiz Halepota, Sarah Khan, Hammad Atif Irshad, Muhammad Arshad

**Affiliations:** 1 Department of Pediatric Surgery, Aga Khan University Hospital Karachi, Stadium Road, Karachi, 74800 Sindh Pakistan; 2 St Jude Children’s Research Hospital Memphis, Karachi, 74800 Sindh Pakistan; 3 Department of Medicine, Aga Khan University Hospital Karachi, Karachi, Pakistan; 4 Medical College, Aga Khan University Hospital, Karachi, Pakistan

**Keywords:** Pediatrics, Vessel extension, Wilms tumor. surgical management

## Abstract

**Objective:**

The aim of this study was to assess management and determine outcomes of renal tumors with inferior vena cava (IVC) and intracardiac (IC) extension in a tertiary care center in Pakistan.

**Methods:**

A retrospective chart review was conducted at the Aga Khan University Hospital, Karachi, Pakistan. All patients from 1 to 18 years of age with renal tumors with intravascular extensions, surgically managed from January 1988 till June 2016, were included. Data was extracted by reviewing medical records, and the tumor details, treatment and outcomes were analyzed.

**Results:**

A total of 18 patients out of the total 61 patients with renal tumors, presented with IVC and/or IC extension, with the majority involving the right kidney. Mean age was 5.9 (SD:4.9) and a female preponderance (56%) was seen. Wilms tumor (77%) was the most common tumor type, with the level of tumor extension into IVC predominantly being below the diaphragm (55.5%). Fourteen patients received preoperative chemotherapy, with tumor regression, seen in 10. Most patients underwent thrombectomy through the renal vein (56%). Regarding outcomes, frequency of mortality and morbidity was 1 and 2, respectively, with 7 patients having no recurrent 5 years post-surgery.

**Conclusion:**

A greater incidence (29.5%) of IVC and or IC Tumor extension was found compared to existing literature, which could likely be due to a higher referral rate to the center. Moreover, this is a single-center study and so a multi-center study is crucial to form an assessment of surgical management in resource-limited settings. Our study is the first from Pakistan on this particular renal tumor presentation. Considering the varying case presentations and surgical techniques used, further studies are needed to standardize surgical management and optimize patient outcomes.

**Supplementary Information:**

The online version contains supplementary material available at 10.1186/s12887-024-05122-1.

## Declarations of interest

none.

## Introduction

Wilms tumor (WT), also referred to as nephroblastoma, is the most prevalent solid organ cancer among the pediatric age group in low- and middle-income countries (LMICs), right after neuroblastoma [1]. With a 97% survival rate in high-income countries (HICs), the numbers decrease when compared to LMICs, in which studies from Africa have found survival ranging around 56% [2, 3]. While other renal tumors include the non-Wilms malignancies such as renal cell carcinoma and rhabdoid tumors. Although there is a paucity of literature evaluating the factors for lesser survival, the gap is likely due to restricted availability of suitable healthcare services, delay in reaching the doctor, decreased ability to provide supportive care and cancer therapy, and a high rate of lost to follow up treatment [4, 5].

Furthermore, WT has the ability to invade blood vessels, and with literature reporting extension into the inferior vena cava (IVC) in 4–10% of cases while in 0.7%, it may extend into the right atrium [1, 6], showing that even intra-cardiac (IC) spread is a possibility, the surgical plans become more complex [7]. Pre-operative chemotherapy in these patients decreases the extension of the tumor thrombus and facilitates resection [8]. On the contrary, the literature suggests this treatment may not cause any significant alteration in postoperative complications [9]. Although they represent a minority of cases but pose a formidable challenge to the surgeon requiring expert surgical and anesthetic care, extensive thrombectomy and in some cases, cardiopulmonary bypass for complete thrombus extraction. Previous studies have also identified the need for further studies on outcomes of caval extension in these tumors [10].

The histological type and stage are the primary determinants of therapeutic success; inadequate tumor excision may result in upstaging [11]. Age, the body’s reaction to treatment, and molecular discoveries are additional variables that affect the result [11]. Although, standardization of renal tumor surgical practice one of the least expensive methods to improve care, the literature is scarce on the management in LMICs [12]. To identify the best management for these patients, we present surgical management and outcomes of renal tumors with intra-caval and intra-cardiac (IC) extension in children operated at our institute. Therefore, the main aim of the study was to assess our institution’s approach to management of renal tumors with intravascular extension in pediatric patients.

## Materials and methods

### Study Design and setting

This retrospective cohort was conducted at the Aga University Hospital (AKUH), Karachi after approval from the Ethical Review Committee (ERC ID: 4341-Sur-ERC-16).

## Selection criteria

All patients below the age of 18 diagnosed with renal tumors with intravascular involvement from January 1988 to June 2016 were included in our study. Patients with renal tumors who did not undergo a surgical procedure at our institution were excluded from the study.

## Data Collection

The medical records were retrieved using the international classification of disease (ICD) code 189.0. Data were retrieved and patient confidentiality was maintained. A questionnaire was made consisting of demographic, tumor, pre-operative, and operative characteristics, which was filled in by the research team members on reviewing the files. The questionnaire can be found in **Supplementary File 1**.

### Statistical analysis

Data was collected and analyzed via Statistical Package for Social Science (SPSS) software version 19. Mean and the standard deviation (SD) were calculated for all continuous variables such as age. For all categorical variables, frequency and percentages have been displayed.

## Results

**Fig. 1:**
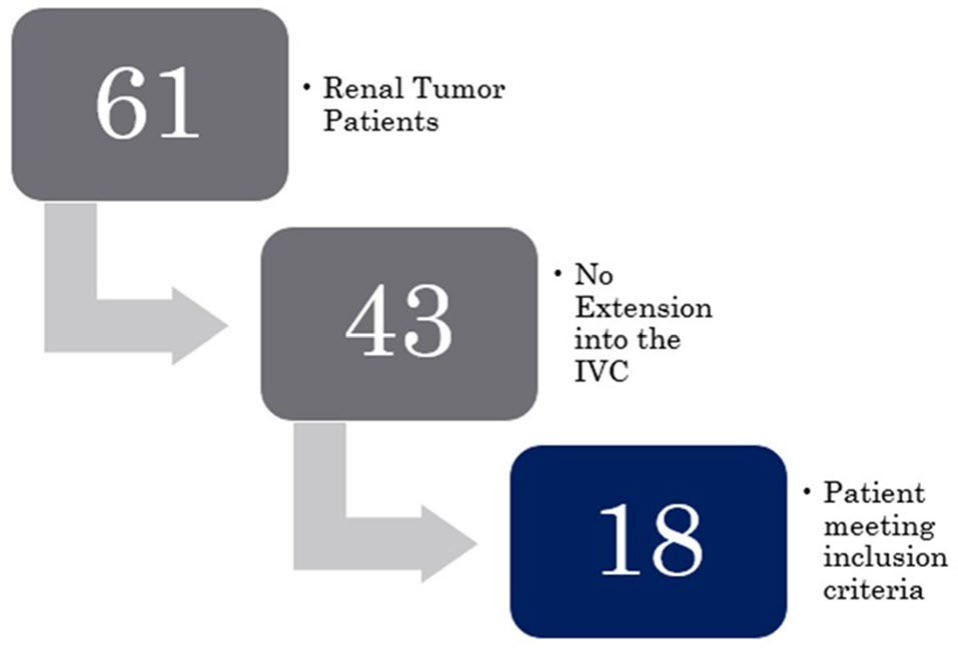
Flowchart of Participant Selection

**Fig. 2:**
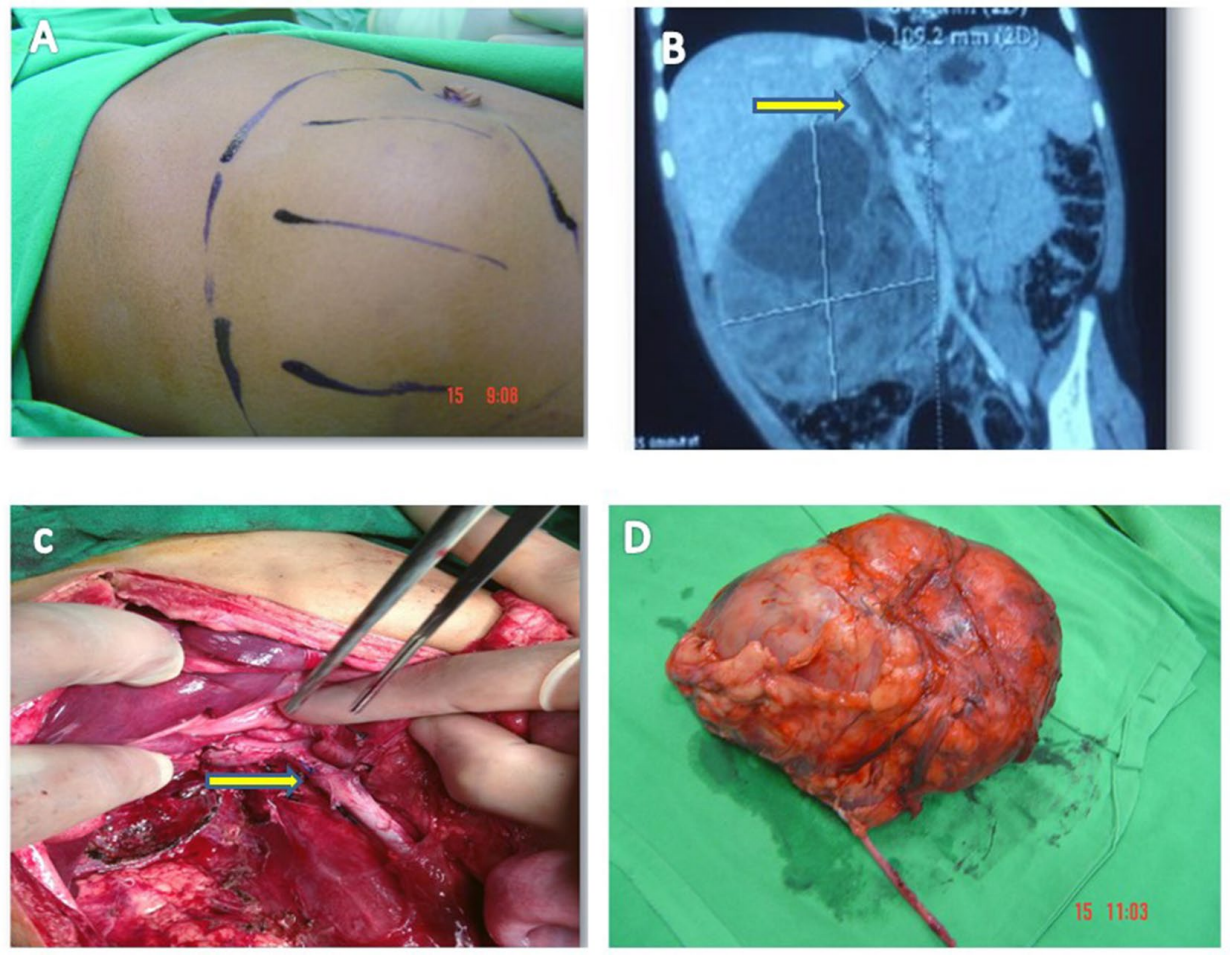
Photographs of a patient 3 years of age who presented with an asymptomatic abdominal mass noticed by mother including: (**A**) Preoperative picture with incision site marked; (**B**) representative coronal contrast-enhanced CT image of the same patient showing right-sided renal tumor along with a tumor thrombus extending into IVC (arrow); (**C**) representative intra-operative picture of the same patient where IVC was opened and thrombus retracted completely followed by repair of the vessel at the selected site (arrow); (**D**) Excised tumor mass

During the study period of 28 years, 61 pediatric patients with renal tumors were managed at our institution as outlined in Fig. 1. Out of these, 18 (29.50%) patients had tumors that showed intra-caval or intra-cardiac extensions. Figure 2 shows a pictorial representation of one of the included cases.

The mean age of these 18 patients was 5.9 years (SD: 4.9). Overall, 10 (55%) patients were females and 8 (44%) were males. In terms of tumor subtypes, 14 (77.77%) had Wilm’s tumor, 3 (16.66%) had Renal Cell Carcinoma and 1 (5.5%) had a rhabdoid tumor. The level of tumor extensions was below the diaphragm in 10 (55.55%), above the diaphragm in 8 (44.44%) while 4 (22.22%) patients showed intra-atrial extension of the tumor. Table 1 shows the sociodemographic and tumor characteristics of the patients.

**Table 1:** Sociodemographic and renal tumor characteristics

Sociodemographic and Tumor Characteristics	
Variable	Frequency (%)/ Mean ± SD
**Age**	5.9 ± 4.9
**Sex** Male Female	8 (44) 10(56)
**Diagnosis** WT RCC Rhabdoid Tumor	14(78) 3(17) 1(5)
**Tumor Laterality** Right Left **Bilateral**	12 (67) 5 (28) 1(5)
**Tumor Stage** 2 3 4 5	2 (11) 8 (44) 7 (39) 1(5)
**Extension** Above Diaphragm Below Diaphragm	8 (44) 10 (56)

WT: wilm’s tumor; RCC: renal cell carcinoma; AD: above diaphragm; BD: below diaphragm

**Fig. 3:**
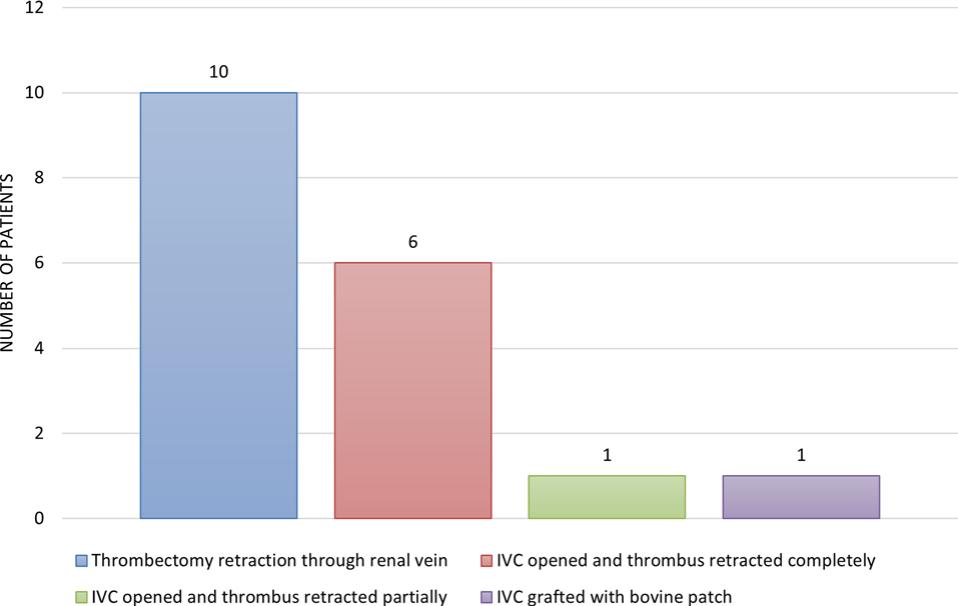
Summary of Surgical Management of all Cases

Figure 3 provides a summary of surgical management of all cases. Of those 14 with Wilm’s tumor, 10 (71%) patients had a below-the-diaphragm extension. The staging of these was 2 with stage 2, 6 with stage 3, and 2 had stage 4 disease. Seven of these patients received preoperative chemotherapy and three underwent thrombectomy. Of the three patients with Renal Cell Carcinoma, all had tumor extension above the diaphragm with two being extending into IC. The child with the above diaphragm extension underwent a failed IVC thrombectomy and was presented with liver metastasis after a year. The other two patients that had an intra-atrial extension, one received preoperative chemotherapy and radiotherapy but was referred to another hospital for further management, while the other had a failed attempted surgical procedure. Only one patient had presented with a Rhabdoid tumor with above-the-diaphragm extension. The patient received preoperative chemotherapy.

Table 2 displays the post-operative details of the included cases. Among the patients with Wilms tumor, six patients reported no recurrence after five years of follow-up while one patient presented with lung metastasis and one with the recurrence of metastatic disease at 1 year of follow up. Two patients were lost to follow up. 2 (14%) patients had an above-the-diaphragm extension and were classified as Stage 4 and 5. Both were treated with pre-operative chemotherapy. One showed no recurrence or relapse after 5 years of follow-up while one of them expired due to refractory disease in a year of treatment. 2 (14%) patients had an intra-atrial extension and were both classified as Stage 3. Both received neoadjuvant chemotherapy followed by thrombectomy. One of these patients left against medical advice (LAMA) and one showed no recurrence after five years of follow-up. All the RCC patients developed metastatic disease. The single rhabdoid tumor patient was expired till follow up.

**Table 2:** Post Operative characteristics of patients

Post-operative Details	
Variable	Frequency (%)
Chemotherapy Yes No Not Applicable	13 (72) 4 (23) 1 (5)
**Radiation** Yes No	7 (39) 11 (61)
**CTCAE Grade** 1 2 3 Not Applicable	10 (56) 4 (22) 2 (11) 2(11)

**CTCAE**: Common Terminology Criteria for Adverse Events

## Discussion

Our study is the first of its kind on a rare entity consisting of vascular and cardiac extension of renal tumors within the pediatric population. We found 2/3rd of the cases in our institution to be of Wilm’s tumor with the level of tumor extension into IVC predominantly being below the diaphragm (55.5%).

Previous literature finds Wilm’s tumor to be associated with intravascular invasion [1, 4]. Renal vein involvement occurs in 10% of the patients and mostly on the right side [1]. Similarly, in our study, 77% of the cases belonged to Wilm’s tumor with around 66% involving the right side.

In most patients, the diagnosis of intravascular extension is made simultaneously with a diagnosis of a tumor through color doppler ultrasonography, triphasic CT, or MRI scan, but 50% of patients present with symptoms of intravascular invasion [1]. On the contrary, in a few studies no patients were presented with symptoms characteristic of IVC extension [4]. In our study, however, none of our patients had any symptoms characteristic of vascular invasion. Moreover, these symptoms were even absent in all patients who had a thrombus extension above the diaphragm or in the heart. This could likely be due to our limited sample size and therefore further studies are needed on this entity to investigate the incidence symptomatology of these patients in Pakistan.

Surgical resection is the definitive treatment for such patients [1]. However, for patients with intra-cardiac extension, cardiopulmonary bypass can be an option; it allows easier access but is an invasive procedure [1]. Fortunately, none of our patients had to undergo CPB. Pre-operative chemotherapy is suggested in literature when the extensive thrombus is identified in the vena cava to reduce the size of the tumor and ease surgical resection [1, 4]. In the past surgical complications in patients with intra-cardiac extension were as high as 73% but recently these have been declining. Various authors have credited this decline in complications to pre-operative chemotherapy [4]. On the other hand, pre-operative chemotherapy should not always be used. It is because it might reduce the size of the tumor, but post-operative complication rates are not significantly different when primary surgery and pre-operative chemotherapy patients are compared regardless of the extent of IVC thrombus [4]. Secondly, pre-operative chemotherapy reduces the accuracy of staging which obscures treatment options, so the cost-benefit analysis is required on a case-by-case basis [4]. Despite this, preoperative chemotherapy is important as it leads to a more favorable stage distribution and considerably fewer tumor ruptures during surgery, making surgeries easier [13]. Other single center studies also found improved overall survival in neoadjuvant therapy resolves the extension [14].

The patient with a Rhabdoid tumor also passed away due to the extension of thrombus into the brain. His brain MRI showed multiple infarcts. Aglaia et al. also had the same complication with one of their patients [1]. Their study suggested that small thrombi from the intra-cardiac extension may have traveled via a minute patent foramen ovale (missed on echocardiograph) up the carotids [1]. Moreover, the extension of a thrombus occurs due to high blood viscosity and low blood flow in the brain veins compared to the arteries [1]. In such cases, early detection is very important as radiotherapy to the brain has favorable outcomes and is treatable [1].

In our study, a high (30%) prevalence of intra-caval and intra-cardiac extension was found among children with renal tumors as compared to 4–10% prevalence of intra-caval and 0.7–3% prevalence of intra-atrial extension reported in the literature. This can be explained by the fact that most of these patients were referred to our institute solely for surgery because of better peri-operative and surgical care and availability of pediatric cardiac surgeons and pediatric cardiac anesthetists at our institute owing to the complexity of operating on renal tumors with IVC extension.

Surgery is the most important part of the treatment. Carefully handling the tumor, proper resection and clean surgery are necessary to minimize surgical complications. Surgical complications in IVC extension patients have an odds ratio of 2.2 by multivariate analysis [15]. Therefore, not only is early diagnosis of renal tumors important, but knowledge about their intravascular extension is also as important. In the early stages of tumor and with favorable histology, Wilm’s tumor can only require chemotherapy to keep patients in complete remission [1]. In extensive lesions, where the heart is involved, patients might need CPB before renal surgery [1]. Furthermore, extension into the renal vein needs pre-operative chemotherapy while brain and bone lesions require radiotherapy before surgery [1].

Management of renal tumors with IVC extension in children is a challenge in resource-limited settings. However, in our study, we found a beneficial role of pre-operative chemotherapy with thrombus regression in 61% of patients receiving preoperative chemotherapy and avoiding the need for cardiopulmonary bypass in all our patients. Appropriate expertise and a multidisciplinary approach can bring optimum results in resource-limited settings.

## Conclusion

Intra-caval and intracardiac extension of renal tumors in children is a surgical challenge in resource-limited centers. Appropriate expertise and a multidisciplinary approach can bring optimum results in resource-limited centers for which more multicenter studies and cancer databases are the need of the hour.

## Electronic supplementary material

Below is the link to the electronic supplementary material.

Supplementary Material 1

## Data Availability

The dataset and its associated materials are available from the corresponding author upon reasonable request.

## Abbreviations

IC: Intracardiac
IVC: Inferior Vena Cava
LMIC: Low- and Middle-Income Country
