# Supplementary material for: Surgical management and outcomes of renal tumors with inferior vena cava extension among children: a single center retrospective study from Pakistan
Source: BMC Pediatr. 2024 Oct 10;24:645. doi: 10.1186/s12887-024-05122-1 (PMC11465680; doi:10.1186/s12887-024-05122-1)
Supplement: Supplementary file 1 — Supplementary Material 1 [file 12887_2024_5122_MOESM1_ESM.docx]

**Surgical Management and Outcomes of Renal Tumors with Inferior Vena Cava Extension among Children: A Single Center Retrospective Study from Pakistan**

***Data Collection Sheet***

- Demographics
  - Age
  - Sex
    - Male
    - Female
- Tumor Characteristics
  - Diagnosis
  - Laterality
    - Right
    - Left
    - Both
  - Stage of Tumor
  - Extension
    - Below Diaphragm
    - Above Diaphragm
- Surgical Management
  - Write the name of the surgical management of the case ____________________
- Post-Operative Characteristics
  - Was chemotherapy received?
    - Yes
    - No
    - Not applicable
  - Was radiation received?
    - Yes
    - No
  - Common Terminology Criteria for Adverse Events (CTCAE) Grade
    - 1
    - 2
    - 3
    - Not Applicable
